# Ocean processes at the Antarctic continental slope

**DOI:** 10.1098/rsta.2013.0047

**Published:** 2014-07-13

**Authors:** Karen J. Heywood, Sunke Schmidtko, Céline Heuzé, Jan Kaiser, Timothy D. Jickells, Bastien Y. Queste, David P. Stevens, Martin Wadley, Andrew F. Thompson, Sophie Fielding, Damien Guihen, Elizabeth Creed, Jeff K. Ridley, Walker Smith

**Affiliations:** 1 Centre for Ocean and Atmospheric Sciences, School of Environmental Sciences, Norwich NR4 7TJ, UK; 2 Centre for Ocean and Atmospheric Sciences, School of Mathematics, University of East Anglia, Norwich NR4 7TJ, UK; 3 California Institute of Technology, Pasadena, CA 91125, USA; 4 British Antarctic Survey, Cambridge CB3 0ET, UK; 5 Kongsberg Underwater Technology, Inc., Lynnwood, WA 98036, USA; 6 Met Office Hadley Centre, Exeter EX1 3PB, UK; 7 Virginia Institute for Marine Science, College of William and Mary, Gloucester Point, VA 23062, USA

**Keywords:** Antarctic continental shelf, Antarctic Slope Front, ocean glider, water mass, climate model, iron fertilization

## Abstract

The Antarctic continental shelves and slopes occupy relatively small areas, but, nevertheless, are important for global climate, biogeochemical cycling and ecosystem functioning. Processes of water mass transformation through sea ice formation/melting and ocean–atmosphere interaction are key to the formation of deep and bottom waters as well as determining the heat flux beneath ice shelves. Climate models, however, struggle to capture these physical processes and are unable to reproduce water mass properties of the region. Dynamics at the continental slope are key for correctly modelling climate, yet their small spatial scale presents challenges both for ocean modelling and for observational studies. Cross-slope exchange processes are also vital for the flux of nutrients such as iron from the continental shelf into the mixed layer of the Southern Ocean. An iron-cycling model embedded in an eddy-permitting ocean model reveals the importance of sedimentary iron in fertilizing parts of the Southern Ocean. Ocean gliders play a key role in improving our ability to observe and understand these small-scale processes at the continental shelf break. The Gliders: Excellent New Tools for Observing the Ocean (GENTOO) project deployed three Seagliders for up to two months in early 2012 to sample the water to the east of the Antarctic Peninsula in unprecedented temporal and spatial detail. The glider data resolve small-scale exchange processes across the shelf-break front (the Antarctic Slope Front) and the front's biogeochemical signature. GENTOO demonstrated the capability of ocean gliders to play a key role in a future multi-disciplinary Southern Ocean observing system.

## 1. Introduction

The Antarctic continental shelf and slope are often considered outposts of ocean research; they are remote, covered in sea ice for much of the year, and small in geographical extent. Mostly poorly mapped, these shallow regions host counter-currents to the largest current on the planet, the Antarctic Circumpolar Current (ACC). Observational studies are mostly restricted to the summer months, whereas Argo floats operating year-round typically provide few profiles south of the ACC. Here, we discuss several areas of science that illustrate the importance of the Antarctic shelf and slope, and the need for further observational and model investigations.

**Figure 1.**
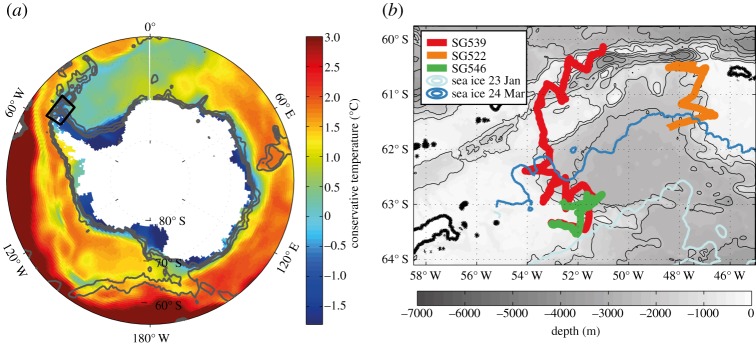
(*a*) Map of Antarctica showing the conservative temperature at 200 dbar from the MIMOC climatology for the month of January [1]. Bathymetric contours (grey lines) are shown at 1000, 2000 and 3000 m to indicate the location of the Antarctic continental slope. Black box indicates the GENTOO study area, shown expanded (*b*) with the three glider tracks. Seagliders Tashtego (SG539, red) and Beluga (SG522, green) were deployed at approximately the same time (January) and location at approximately 63.5° S, 53° W. Seaglider Flying Pig (SG546, orange) was deployed two weeks later in the Powell Basin to survey the continental slope of the South Orkney Islands. Cyan line indicates the sea ice edge (15% concentration) at the beginning of the glider deployment and blue line indicates the ice edge when Tashtego was recovered in March.

The Antarctic continental shelf (figure 1) is typically about 500 m deep, and the distance from the Antarctic ice sheet or coastline to the shelf break varies from tens (in East Antarctica or the West Antarctic Peninsula) to hundreds of kilometres (in the Ross or Weddell Seas). The continental slope is steep and poorly mapped, but where bathymetry data exist, deep glacial channels and canyons are often evident. Polynyas (areas of open water in otherwise sea ice-covered regions) frequently occur adjacent to the Antarctic continent, where strong katabatic winds blow from the continent and advect the ice northwards. The strong winds and low air temperatures lead to large heat loss from the ocean, so new sea ice forms. The resulting brine rejection is the primary cause of dense water formation on the continental shelf, which subsequently spills off the shelf to form Antarctic Bottom Water and plays a key role in the regulation of global climate [2]. The water masses on the Antarctic continental shelf also provide heat to the underside of ice shelves [3]. A change in either the volume transport or the temperature of water flowing beneath an ice shelf can lead to enhanced ice mass loss from Antarctica.

Water mass properties on the Antarctic continental shelf and slope are poorly observed, particularly in winter because of extensive sea ice cover. The Synoptic Antarctic Shelf–Slope Interactions (SASSI; [4]) study for the International Polar Year was a concerted effort involving 13 nations to make oceanographic measurements around the continent, but the resulting sections or moorings are often thousands of kilometres apart.

January potential temperature at 200 dbar (figure 1) from a climatology of historical observations [1] clearly distinguishes cold shelf waters from relatively warm open ocean waters to the north. The boundary, known as the Antarctic Slope Front, is a near-circumpolar front with downward-sloping isopycnals towards the continent and an associated westward jet, the Antarctic Slope Current. The warm offshore water mass at this depth is Circumpolar Deep Water that provides a large potential source of heat where it crosses onto the shelf. High-resolution ocean model experiments [5] have recently highlighted the important role of eddies in the Slope Front for determining the exchanges of dense cold water from the shelf and warm water from offshore, thus affecting ice melt.

In addition to the remote nature and frequent sea ice cover of the Antarctic continental shelf, the intrinsically small scale of its oceanographic processes poses challenges. The open ocean Rossby radius at these latitudes is typically less than 10 km, and can be as small as 1–2 km on the continental shelf. This determines the typical length scale of eddies, jets and meanders, and means that numerical models designed to be eddy resolving need to have a particularly high resolution, and that observations across the continental shelf and slope need to be closely spaced.

## 2. Representation of Antarctic shelf and slope processes in climate models

The processes on the Antarctic continental shelf and slope are important for global climate and biogeochemical cycles, but steep bathymetry, small spatial scale of fronts and eddies, and complex interactions between atmosphere, sea ice, meteoric ice and water masses combine to make this region particularly challenging for climate models. Considerable efforts are being expended at climate modelling centres worldwide to address these issues. Can the latest generation of climate models adequately represent the water masses around Antarctica?

**Figure 2.**
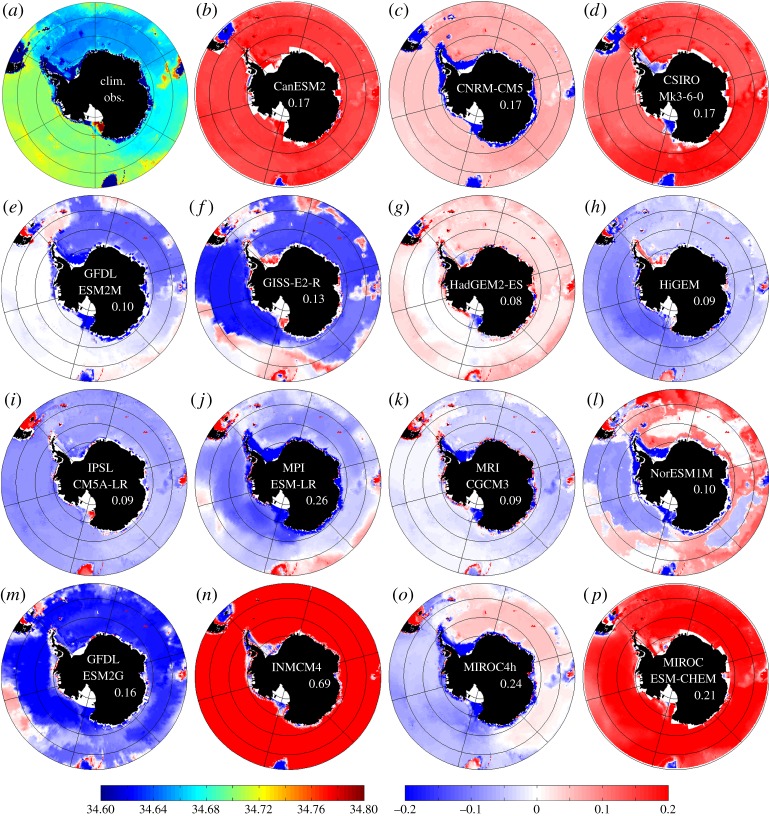
Mean bottom practical salinity of the World Ocean Circulation Experiment (WOCE) climatology (*a*) and mean bottom salinity difference (model-climatology) (*b*–*p*) for a selection of 15 CMIP5 climate models for winter (August) 20 year mean. Left colour bar corresponds to the climatology, right colour bar to the differences model-climatology. Numbers indicate area-weighted root mean square differences (RMSD) for all depths between the model and the climatology. Mean RMSD is 0.18.

Heuzé *et al*. [6] assessed 15 of the coupled climate models available from the fifth Coupled Model Intercomparison Project (CMIP5). Temperature and density of the bottom waters in the abyssal Southern Ocean were compared with climatological values from historical data. Here, we assess the salinity at the seabed in the same climate models. Means of bottom values were computed for the month of August from 1986 to 2005 of historical climate model runs and compared with the observed climatology. Figure 2 illustrates the spatial distribution of the salinity differences from climatology for each of the models. For the Southern Ocean as a whole, there is no consistency between models; they show salinity biases of ±0.2 which are significant in a region where much of the stratification is salinity-determined. Salinity biases have large regional differences within each model (indicated by large values for the RMS differences), which suggests that eliminating such biases will not be straightforward.

**Figure 3.**
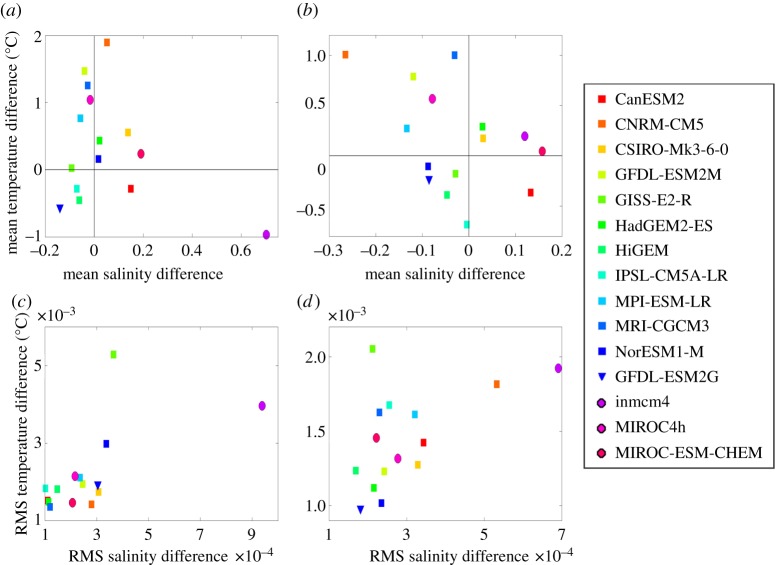
Area-weighted mean (*a*,*b*) and root mean square (*c*,*d*) property differences for each climate model compared with climatology (model minus observations) for the deep Southern Ocean (*a*,*c*; bathymetric depth greater than 2000 m) and the shelves (*b*,*d*; bathymetric depth less than 2000 m). The RMS differences have been calculated after first removing the mean bias for each model. *Z*-level models have square symbols; the isopycnal coordinate model a triangle; and the sigma coordinate models have circles. All calculations are for the Southern Ocean between 80° S and 50° S.

One focus of attention to improve the global deep water mass characteristics in climate models might be to better simulate the ocean–atmosphere–ice processes on the continental shelves of Antarctica. The seabed properties in the Southern Ocean are determined by the mechanisms that replenish the dense abyssal water. Observations suggest that the primary mechanism is the spilling of dense, cold, saline shelf water off the continental shelf. The water descends the continental slope entraining ambient water in the process, before entering the abyssal ocean where it becomes known as Antarctic Bottom Water. However, most climate models are unable to simulate this formation process. The ‘successful’ models (in terms of their representation of ocean properties in the abyssal ocean) are those that form dense water by open ocean deep convection [6], thus incorrectly representing key processes. We now consider separately the salinity of the waters on the Antarctic continental shelf and in the abyss (figures 2 and 3) to shed light on these processes.

Figure 3*a*,*b* summarizes for all models the area-weighted mean bottom temperature and bottom salinity differences (model minus observations) for the deep Southern Ocean (bathymetric depth greater than 2000 m) and the Antarctic shelves (bathymetric depth less than 2000 m). For each domain, we remove these biases prior to calculating the root mean square (RMS) differences shown in figure 3*c*,*d*. Models exhibiting very different RMS and mean differences are those with highly spatially variable biases. More than half of the models exhibit a fresh bias on the shelf and slope. Some models demonstrate a fresh bias but with very localized saline biases, resulting in average high-density biases on the shelf. These localized highly saline and dense water masses may be able to sporadically escape the continental shelf and contribute to replenishing the Antarctic Bottom Water, but such an intermittent process is difficult to detect in monthly climate model output. This localized formation of dense water could be a very effective way in which a climate model could replenish the abyss, even if it is not accurately simulating the processes occurring in the real ocean.

In general, the models that have large temperature or salinity biases in the abyss also have large biases on the continental shelf; models mostly (although not always) lie in the same quadrants in figure 3*a*,*b*. There are no significant differences between the numbers of models in each of the four quadrants; biases are seemingly random. Models that have larger temperature biases do not necessarily have large salinity biases, and vice versa. Models can have large mean biases, but small de-biased RMS differences, indicating that the whole of the Southern Ocean is uniformly biased in those models. There is no apparent relationship between model fidelity and coordinate scheme or resolution (the bottom row of models in figure 2 are sigma or isopycnal coordinate models and the others are *z*-coordinate models; the coordinate systems are indicated by different symbols in figure 3). The lack of any particular patterns in the characteristics of the models in figure 3 confirms that improving deep water mass characteristics is not likely to require the same solution in any two models; the interplay of the climate model components is too complex.

The model output in figure 2 represents winter conditions, when the sea ice is at a maximum and salinity on the Antarctic continental shelf might be expected to be high. The climatology (figure 2*a*) is biased towards summer observations when ice is melting. We might expect a seasonal saline bias in the modelled water masses at the seabed on the continental shelves, however this would be associated with a cold bias. Too saline water masses on the continental shelf may be a result of too much sea ice formation in the models, generating large amounts of brine that sinks to the seabed.

The properties of the water masses on the Antarctic continental shelf are poorly simulated, but there is no consistent behaviour in the models studied, in the formation of either dense shelf water or abyssal water. Some simulations are too fresh and others too saline, and the biases on the shelf are not always the same in the deep Southern Ocean. Some models probably produce dense water through unrealistic processes. Does it matter that climate models are unable to represent these water masses? It does, especially if we need to accurately predict likely future melt rates and stability of Antarctic ice shelves and ice sheets, because the relatively warm ocean provides the heat source for ice shelf basal melt. If the climate models form too much thick sea ice that is not advected away, then this prevents heat from leaving the ocean [7]. The formation of Antarctic Bottom Water is a crucial part of the coupled climate system, yet the climate models are unable to both create and export water of correct characteristics.

Coastal and open ocean polynyas are one of the processes that need to be better represented in climate models. Although they have a relatively small geographical extent compared with sea-ice-covered regions, polynyas are the sites of the most rapid and extensive changes in water mass properties through loss of heat to the atmosphere and brine rejection through sea ice formation. The next-generation climate models are evaluating ice shelf cavities [8], which may improve the shelf water properties. However, the fundamental problem of continental shelf edge flows can be resolved only with high horizontal and vertical resolution. Even global model resolutions of 1/12° are insufficient to be eddy permitting at high latitudes, so alternative techniques may be required. Such methods include two-way embedded high-resolution models [9], or targeted irregular mesh models [10].

## 3. Seaglider observations of Antarctic processes on the shelf and slope

The complexity and small spatial scale of the processes on the Antarctic continental shelf and slope are not only a challenge for numerical models, but they also pose a challenge for observational oceanography and in the design of the Southern Ocean Observing System (SOOS). Regional and idealized models have suggested that the space and time scales of variability on the Antarctic margin may be as small as O(1 km) and a couple of days [5,11,12]. These scales are not amenable to study by ship or moorings. One emerging technology is the ocean glider, an autonomous underwater vehicle (AUV) able to profile the ocean for months at a time [13]. Without a propeller, gliders are more energy efficient than other AUVs, and data can be transmitted back to the user each time the glider surfaces (typically every 4 h for dives to 1000 m depth, with dives separated horizontally by a few kilometres). This is a great advantage in a region that is both inaccessible for ships and covered with sea ice that can advance or break up unpredictably within days; the loss of a glider does not mean the loss of the entire dataset.

Gliders can carry a range of sensors to equip them for the interdisciplinary challenges of the twenty-first century. The standard sensors presently include temperature, salinity, dissolved oxygen, chlorophyll fluorescence and optical backscatter. Recently developed sensors include photosynthetically active radiation, acoustic backscatter for zooplankton/micronekton, *p*(CO_2_) and pH. Considerable effort is being expended worldwide to design and develop small, lightweight sensors that do not have a large energy requirement, particularly for biogeochemistry and ecology. In addition, gliders are being deployed in ever more challenging environments, such as beneath seasonal ice cover using acoustic navigation [14].

Recently, gliders have begun to be used in polar regions; we discuss one such deployment, to highlight the insights that may be gained, particularly at the Antarctic shelf break. In January 2012, three Seagliders were deployed to the east of the tip of the Antarctic Peninsula (figure 1) as part of the Gliders: Excellent New Tools for Observing the Ocean (GENTOO) project. The goal was to survey the continental shelf and slope of the Weddell Sea to assess the spatial and temporal variability of the water masses and the Slope Front. Extensive sea ice in early 2012 meant that the majority of the Seaglider sections crossed the slope in the Powell Basin and progressed gradually northward for recovery in March.

**Figure 4.**
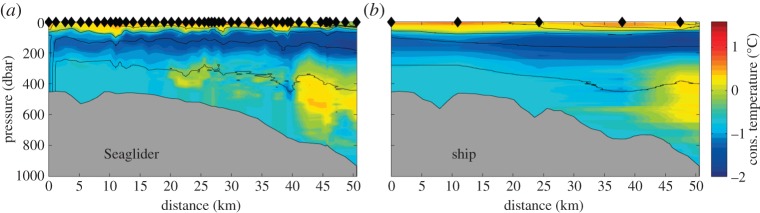
Comparison of near-simultaneous glider (*a*) and ship-based (*b*) conservative temperature sections across the Antarctic continental slope into the Weddell Sea. Locations of surface measurements are indicated by the black diamonds on the upper axis. Bathymetry is shown in grey. Potential density contours are shown every 0.1 kg m^−3^ as black lines.

Comparing the southernmost Seaglider section with a relatively closely-spaced ship-based conductivity–temperature–depth (CTD) section (figure 4) occupied at the same time, the additional small-scale structure seen by the glider is apparent. A CTD station spacing of 10 km, which would typically resolve the submesoscale structure in mid-latitudes, is problematic at this latitude. There is a suggestion of some cold water in the core of the Slope Front (40 km along the section at approx. 400 m), but this is inadequately resolved. The glider provides 10 times the number of profiles, and this subsurface cold water is identified on many dives by two independent gliders. This cold water is immediately adjacent to the core of the Warm Deep Water (a warm, more saline Weddell Sea water mass originating from Circumpolar Deep Water circulating in the Weddell Sea) marking the offshore limit of the front. It is isolated from the cold water above in the Winter Water layer, and the water column is statically stable. It is possible that it represents the remnant of a diapycnal mixing process that has brought the Winter Water downward by 200 m. However, the most likely origin is from cold, shelf waters that have entered the Antarctic Slope Current further south in the Weddell Sea, and been advected northwards. Much of the small-scale structure seen in the glider data is associated with internal waves, particularly on the sharp boundary at the base of the temperature-minimum layer (Winter Water). However, the small subsurface lenses of warmer water at 20–35 km indicate that Warm Deep Water has crossed the Slope Front onto the continental shelf. Such bidirectional cross-frontal and cross-slope exchange processes are likely to be important for the exchange of nutrients, larvae and carbon between the open ocean and continental shelf.

**Figure 5.**
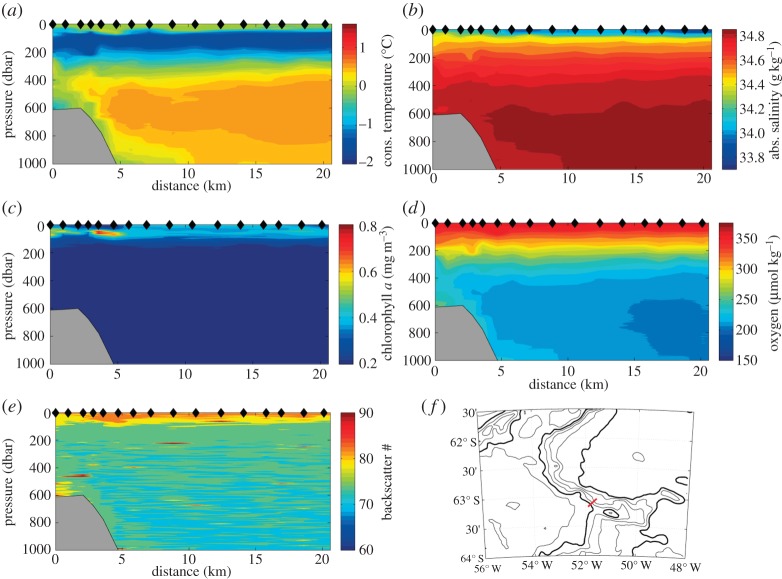
An example Seaglider section from the continental shelf (distance 0 km) into Powell Basin (distance 20 km) of (*a*) conservative temperature (°C), (*b*) absolute salinity (g kg^−1^), (*c*) chlorophyll *a* concentration (mg m^−3^) calculated using a factory calibration and not calibrated against *in situ* phytoplankton assemblages, (*d*) dissolved oxygen (μmol kg^−1^), and (*e*) optical backscatter signal strength (uncalibrated counts). The locations of glider surfacings are indicated as black diamonds on the upper axes. The seabed is shown in grey deduced from General Bathymetric Chart of the Oceans (GEBCO) atlas data. (*f*) The location of the section is indicated in red, together with bathymetric contours every 500 m. The 1000 and 3000 m contours are shown in bold.

Figure 5 shows an example Seaglider section across the continental slope of the Powell Basin, downstream of the section shown in figure 4. At the seabed, we see the cold water core (figure 5*a*) that has spilled off the continental shelf further south in the Weddell Sea, and is now being exported from the Powell Basin to form what will eventually be termed Antarctic Bottom Water. It is relatively rich in dissolved oxygen (figure 5*d*) compared with the Warm Deep Water above it, indicating that it was recently ventilated. Optical backscatter (figure 5*e*) is also slightly elevated in this cold layer, suggesting that the flow here is bottom-enhanced, sufficiently fast to suspend sediments in a thick nepheloid layer. The V-shaped isotherms and isohalines that indicate the location of the Antarctic Slope Front are apparent at an along-track distance of 102 km. Chlorophyll *a* concentrations show a consistent pattern of more chlorophyll *a* near the Slope Front and on the continental shelf than in the open Weddell Sea. This suggests greater phytoplankton growth on the shelf, perhaps owing to enhanced surface stratification or to sedimentary sources of iron.

## 4. Antarctic shelf influence on iron concentrations

**Figure 6.**
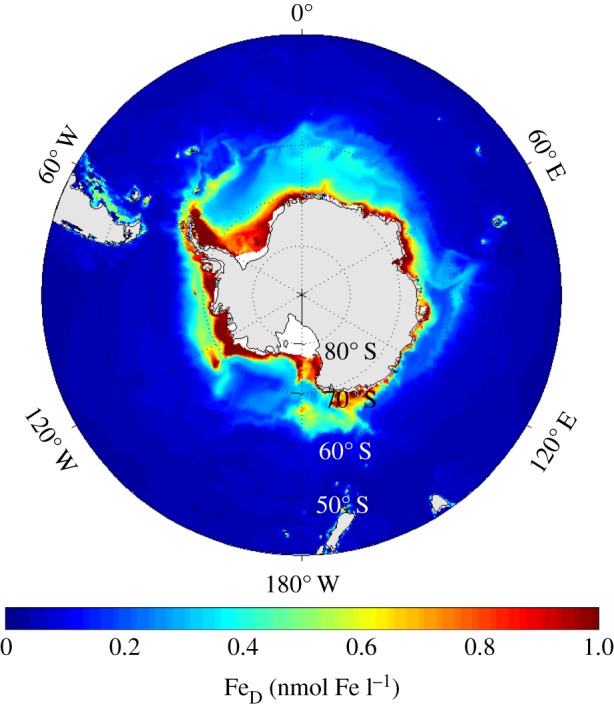
Annual mean distribution of dissolved iron (nmol l^−1^) in the ocean mixed layer of an iron-cycling model driven by the likely sources of iron (dust, icebergs and sedimentary) [19].

The Southern Ocean plays a significant role in the drawdown of anthropogenic CO_2_, yet this remains poorly constrained [15]. It is now known as a region where the limiting trace element for primary productivity is iron [16]. One source of iron is from dust from continental weathering, deposited in the ocean after atmospheric transport or glacial melt; inputs are generally small in the Southern Ocean owing to its distance from non-ice-covered continental land masses. Icebergs also transport iron from the continental margin and have been shown to enhance productivity [17]. However, the most significant iron source to the Southern Ocean is likely to be the iron-rich sediment on the continental shelves and shallow plateaus surrounding the continent and the sub-Antarctic islands. Numerical ecosystem modelling studies of the influence of iron [18] have had some success in capturing the spatial distribution in productivity. A study of the sensitivity of the productivity to these iron sources [19] uses a much simpler iron-cycling model embedded in an eddy-permitting ocean circulation model. It has three iron reservoirs: dissolved iron, iron within the living plankton and sinking or particulate iron. The model's predicted dissolved iron distribution (figure 6) demonstrates the importance of the Antarctic continental shelves as an iron source. Dissolved iron concentrations are highest in the mixed layer around Antarctica. Comparison with figure 1 confirms the large mixed layer iron concentrations in the continental shelf regions inshore of the Antarctic Slope Front, but also reveals that the iron is being exported off the shelf. The model output suggests that sediment provides some 90% of the usable iron in the Southern Ocean and icebergs the remaining 10%. Additional localized sources include dust deposition (e.g. downwind of Patagonia). Sediment fluxes on the Antarctic continental shelf and slope must be able to cross the barrier of the Antarctic Slope Front in order to fertilize the open ocean, emphasizing the importance of the cross-slope processes in transporting nutrients as well as biota. Icebergs provide one route in which such nutrients may cross such a boundary. The processes of eddy overturning and frontal instability [5], as observed by the glider data discussed in §3, may also play a role. Icebergs and shelf water both contribute sources of iron to the exceptional productivity seen in the Scotia Sea within an otherwise unproductive iron-limited Southern Ocean. The high-resolution (1/12°) model in which iron cycling is embedded is able to simulate such processes. However, it is important to note that such a high resolution is not yet possible in the Earth system models, and therefore the role of the Antarctic continental shelf in global scale carbon cycling may remain underestimated.

## 5. Conclusion

**Figure 7.**
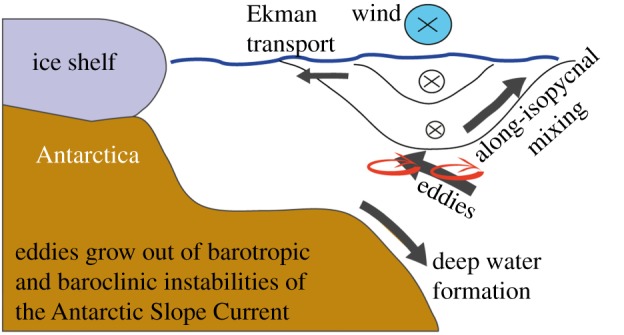
Schematic to summarize the principal processes acting at the Antarctic continental shelf break that are involved in the exchange of water masses, heat, freshwater, nutrients and/or biota.

We have shown examples to illustrate the importance of the Antarctic continental slope and shelf for climate and for biogeochemistry, highlighting that a relatively small area can have a disproportionately large influence on global ocean properties. Figure 7 summarizes schematically the processes at the Antarctic shelf break that are important for cross-slope exchange of heat, freshwater, nutrients such as iron or larvae and other biota. It is clear that we are only just beginning to have the tools available to understand these polar processes, let alone to parameterize them in a suitable way for their inclusion in global Earth system models. Ocean gliders offer great potential for observing Antarctic processes; they can undertake multi-disciplinary missions for some months without a ship being in attendance. The Seaglider results shown here identified ocean features that could not feasibly have been studied by any other means; furthermore, such eddy processes are likely to be common all around the Antarctic margin. It is likely therefore that gliders can have an important role in the future SOOS for continental shelves and slopes, along with other new technologies such as miniature sensors carried by marine mammals. Better understanding of the processes must be obtained concurrently with the development of new modelling techniques to improve the representation of Southern Ocean water masses in climate models.
